# Non-Invasive Measurement of Serum Haemoglobin for the Detection of Postpartum Anaemia: A Prospective Observational Study

**DOI:** 10.3390/jcm15072483

**Published:** 2026-03-24

**Authors:** Gabriel Honnef, Barbara Hallmann, Michael Eichlseder, Michael Eichinger, Philipp Zoidl, Martina Kollmann, Paul Zajic, Nikolaus Schreiber, Martin Rief, Helmar Bornemann-Cimenti

**Affiliations:** 1 Department of Anaesthesiology and Intensive Care Medicine, Medical University Graz, Auenbruggerplatz 5, 8036 Graz, Austria; gabriel.honnef@medunigraz.at (G.H.);; 2 Department of Obstetrics and Gynaecology, Medical University of Graz, Auenbruggerplatz 14, 8036 Graz, Austria

**Keywords:** anaemia, postpartum, non-invasive haemoglobin measurement, haemoglobin, haemorrhage, childbirth

## Abstract

**Background/Objective**: The primary aim of this study was to establish a clinically relevant cut-off for detecting postpartum anaemia using non-invasive haemoglobin measurement, and to compare the non-invasive method with laboratory haemoglobin testing. This study was conducted as a prospective observational study at a single centre. Pregnant women giving birth vaginally or by caesarean birth at a university hospital were included in this study. **Methods**: We measured haemoglobin by non-invasive and laboratory means at delivery room discharge and after childbirth in the operation room. We then calculated a clinically relevant cut-off for detecting postpartum anaemia using the non-invasive measurement method. The main outcomes were the invasively and non-invasively measurements of haemoglobin and the correlation between the two measurements. **Results**: In total, 466 complete measurement pairs from 323 women were included, and 179 (38.4%) laboratory measurements were found to be anaemic (haemoglobin value < 11 g dL^−1^). Maximising specificity while maintaining a minimum sensitivity of 80%, we identified a cut-off of 13.75 g dL^−1^, which achieved a sensitivity of 81.0% and a specificity of 54.7%. The NPV at this threshold was 82.2%, while the PPV was 52.7%. The mean difference between measurements was found to be +2.3 g dL^−1^ (CI 95% 2.16 to 2.43). **Conclusions**: Non-invasive haemoglobin measurement did not sufficiently detect anaemia compared to laboratory measurement values in the setting of early postpartum women, even after adjusting for bias. However, the proposed cut-off could potentially aid healthcare providers in low-resource situations.

## 1. Introduction

Knowledge of a patient’s serum haemoglobin (Hb) level is of great importance in many areas of medical practice. This is particularly true in pregnant women due to the high incidence of relevant bleeding associated with delivery. Significant blood loss during childbirth is common, and the rate of postpartum haemorrhage (PPH) worldwide is still relatively high [1,2,3]. Postpartum haemorrhage impacts approximately 5% of women following delivery [4]. Nearly one quarter of all maternal deaths worldwide are linked to postpartum haemorrhage. In many low-income nations, it remains as the leading cause of maternal mortality [2,4].

The definition of postpartum anaemia varies between different national and international guidelines, ranging from a Hb cut-off of <10 mg dL^−1^ to <11 mg dL^−1^—the latter is used by the World Health Organization (WHO). The prevalence of anaemia after childbirth has remained high in both developed (22–50%) and developing (50–80%) countries over the last few decades, and postpartum anaemia has been shown to have serious implications on health and maternal well-being [1,3,5,6].

Determining the severity of maternal postpartum anaemia based on estimated blood loss is often inadequate [7]. Clinical estimation, which relies on vital signs and a visual assessment of blood loss, is a straightforward approach, but it may frequently underestimate the actual severity of postpartum bleeding [8,9].

Standard methods of measurement require direct blood sampling; they are relatively invasive, painful, time-consuming, and therefore not always feasible (i.e., in developing countries, out-of-hospital births).

However, anaemia typically has a straightforward treatment approach; therefore, missed cases pose an unnecessary risk of increased morbidity and mortality. A more readily available diagnostic test could thus improve treatment in many birth settings.

From a screening perspective, diagnostic strategies should prioritise high sensitivity to minimise false-negative results [10]. In a postpartum context, the under-detection of anaemia may delay treatment and impair maternal recovery and well-being [3,11].

In recent years, non-invasive Hb measurement techniques utilising finger probes (SpHb) have been introduced. Numerous studies have been conducted to evaluate the accuracy of these devices by comparing their results with complete blood count (CBC) data across various clinical settings, including operating rooms, critical care units, and preoperative and blood donor clinics [12,13,14,15].

Nevertheless, the diagnostic performance of SpHb varies across clinical settings and patient populations, as demonstrated in systematic and clinical investigations [13,14,15].

Reduced accuracy has also been reported under conditions of altered peripheral perfusion and haemodynamic instability [16,17]. As such, physiological alterations are common in the immediate postpartum period, and the reliability of spectrophotometric haemoglobin estimation warrants specific evaluation in this context, particularly as low sensitivity has recently been described in postpartum women [18].

However, to date, limited studies have evaluated the utilisation of these devices in a postpartum setting and, to the best of our knowledge, no cut-off values for the detection of postpartum anaemia using SpHb measurements have been established so far.

The primary objective of this study is to evaluate the effectiveness of SpHb as a screening tool for anaemia in the postpartum period by identifying a clinical relevant SpHb thresholds.

Previous studies evaluating non-invasive haemoglobin monitoring have explored diagnostic performance across different thresholds and reported screening sensitivities frequently in the range of approximately 80%, although clinically established cut-off values remain lacking [18,19].

With reliable cut-off values, SpHb measurement could serve as an additional safety net for the detection of postpartum anaemia, particularly in patients who may not routinely undergo invasive Hb testing, such as those in developing countries or following out-of-hospital births. In these settings, invasive blood testing is often neither feasible nor practical. A non-invasive measurement method could help midwives and other healthcare providers identify women at risk of anaemia and refer them for further testing.

Furthermore, non-invasive measurements could reduce the burden of repetitive venous sampling and contribute to the reduction in stress and pain during the vulnerable postpartum period.

## 2. Materials and Methods

This article was written according to the STARD 2015 and STROBE guidelines.

### 2.1. Study Design and Patient Population

This project was conducted as a prospective observational study at the Department of Obstetrics and Gynaecology at the Medical University Clinic of Graz, Austria. Women who planned to give birth vaginally or by caesarean delivery between 7 September 2023 and 30 June 2024 were screened for inclusion. There was no public and patient involvement (PPI) in the planning of this study.

Due to staffing and logistical constraints, non-invasive SpHb measurements were mainly performed during regular working hours (7 a.m.–3 p.m.). As paired measurements were required for diagnostic accuracy analysis, participants without an available SpHb value could not be included in the final study population. This restriction was based on logistical feasibility rather than clinical characteristics.

### 2.2. Ethics Approval and Consent to Participate

This sub-study was prospectively registered as part of the “The Incidence of Hyperfibrinolysis During Vaginal Delivery and Caesarean birth” study on ClinicalTrials.gov (ID: NCT05975112). The Medical University of Graz’s ethics committee granted ethical approval (decision number: 35-151 ex 22/23) on the 17 March 2023.

Potentially eligible women were informed about the study’s objectives, intervention, and protocol by an anaesthesiologist during their routine check-ups or in the delivery room if feasible. They were then asked to provide written informed consent. Participants retained the right to withdraw their consent for data usage at any point, without providing a reason, in accordance with Austrian and European legal standards. Written informed consent was collected from all patients included in the study. Start of participant data collection was 7 September 2023.

### 2.3. Inclusion and Exclusion Criteria

The inclusion criteria were (1) age ≥ 18 years at the time of screening; (2) planned caesarean or vaginal birth; and (3) childbirth during normal working hours (7 a.m.–3 p.m.) due to laboratory and staff capacity. The exclusion criteria were (1) unavailability of suitable laboratory Hb measurement; (2) patient refusal; and (3) perfusion index < 2.

### 2.4. Measurements and Data Management

CBC collection to analyse Hb values was performed according to local standard operating procedures via venous sampling by a healthcare professional, both at delivery room discharge (within 2 h after vaginal birth or within 3 h after caesarean birth) and in the operation room during caesarean birth, if applicable. Laboratory Hb was analysed by the institution’s laboratory facilities using Sysmex XN-1000 (Sysmex K.K., Kobe, Japan). Anaemia was defined as a Hb value < 11 g dL^−1^. Patients without a suitable laboratory Hb measurement were not included in the study population.

SpHb measurements were performed at delivery room discharge and/or during caesarean birth (within 30 min after childbirth), both accompanying CBC sampling.

SpHb was measured directly after each CBC sampling using a Rad-67™ Spot-check Pulse CO-Oximeters^®^ (Masimo corporation, Irvine, CA, USA) by specially trained healthcare providers. This device utilised a finger probe based on multi-wavelength co-oximetry for the spectrophotometric estimation of patients’ Hb. The sensor was placed on one of patients’ digits in a recumbent position. After 60 s, SpHb values were recorded if the perfusion index (PI) was >2. Measurements with a PI < 2 were excluded and a second attempt with a different finger was conducted.

PI values ranged from 0.02 (indicating weak perfusion) to 20 (indicating good perfusion) and reflected the ratio of pulsatile to non-pulsatile blood flow at the location where the SpHb sensor was applied. PI values < 2 have been reported to decrease the accuracy of SpHb measurements [16,17].

Additional data collected included patient demographics (age, parity, body mass index), gestational age at delivery, mode of delivery, CBC results, SpHb, perfusion index, and time of SpHb measurement.

AI-assisted proofreading was used to improve grammar and clarity. All scientific content remains the sole responsibility of the authors.

### 2.5. Statistical Analysis

Patient characteristics were summarised as means and standard deviations (SD) for continuous variables, or as counts and percentages for categorical variables, as appropriate. To assess the reliability of SpHb measurements in detecting anaemia, sensitivity and specificity were calculated using CBC values as the reference standard. A receiver operating characteristic (ROC) analysis was performed to evaluate the predictive capability of SpHb for identifying anaemia. As this investigation was conducted as an exploratory sub-study on diagnostic accuracy embedded within a larger prospective project, no formal a priori sample size calculation targeting sensitivity estimation was performed. Instead, recruitment continued over the predefined study period in order to obtain the largest feasible number of paired measurements.

The primary objective was to identify a clinically relevant cut-off by maximising specificity while maintaining a minimum sensitivity of 80%, as suggested by similar studies in other settings [19]. Furthermore, we determined the optimal cut-off value using Youden’s J statistic to select the best threshold for detecting postpartum anaemia based on non-invasive SpHb measurements. Confidence intervals for the AUC were calculated using DeLong’s method [20].

Finally, the difference between the two measurement methods was analysed using Bland–Altman analysis, which included calculating the mean deviation and limits of agreement (mean deviation ± 2 × standard deviation). Precision was reported using the mean, measurement differences, and limits of agreement [21].

All data were documented using Microsoft Excel 2016 (Microsoft, Redmont, WA, USA). Analyses were conducted using R (R Foundation for Statistical Computing, Vienna, Austria).

## 3. Results

### 3.1. Descriptive Data

**Figure 1:**
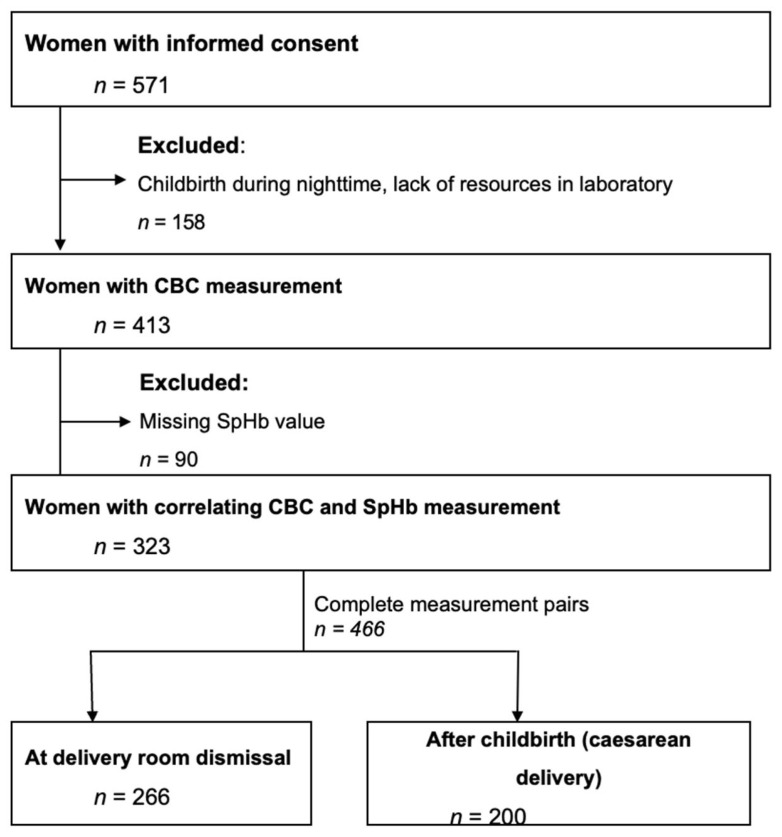
Flow chart. CBC = complete blood count; SpHb = non-invasively measured haemoglobin.

A total of 571 women gave their written consent to participate in this study during a routine prenatal check-up or on the day of delivery. 248 patients were excluded because of missing data. The final analysis was conducted with 466 measurement pairs from 323 patients (Figure 1).

A total of 179 (38.4%) of CBC measurements were found to be anaemic. The mean patient age was 32 (SD 5.2). Most included women gave birth by caesarean delivery (84.8%). Full characteristics of the 466 measurement pairs are presented in Table 1.

Serum haemoglobin ranged from 7.4 to 15.1 g dL^−1^, with a mean CBC Hb of 11.2 (SD 1.2) g dL^−1^ [at delivery room dismissal 11.2 (SD 1.3) g dL^−1^; after caesarean delivery 11.2 (SD 1.2) g dL^−1^]. SpHb ranged from 10.2 to 19.3 g dL^−1^, with a mean SpHb of 13.4 (SD 1.4) g dL^−1^ [at delivery room dismissal 13.6 (SD 1.4) g dL^−1^; after caesarean delivery 13.3 (SD 1.5) g dL^−1^].

The sensitivity of SpHb to detect anaemia in postpartum women was 0.05 (95% CI 0.03–0.09), while the specificity was 0.99 (95% CI 0.97–0.99), resulting in an accuracy of the device to detect anaemia of 0.63 (95% CI 0.59–0.67). There was no significant difference in the accuracy of the device depending on the time of measurement (accuracy after caesarean delivery 0.67 (95%CI 0.60–0.73) vs. at delivery room dismissal 0.60 (95% CI 0.54–0.66) (Table 2).

### 3.2. Primary Outcome

By maximising specificity while maintaining a minimum sensitivity of 80%, we identified a cut-off of 13.75 g dL^−1^, which achieved a sensitivity of 81.0% and specificity of 54.7%. The NPV at this threshold was 82.2%, while the PPV was 52.7%. With this cut-off, 145 women with anaemia would have been detected by non-invasive SpHb measurement while 34 would have been missed (Table 3).

### 3.3. Secondary Outcomes

**Figure 2:**
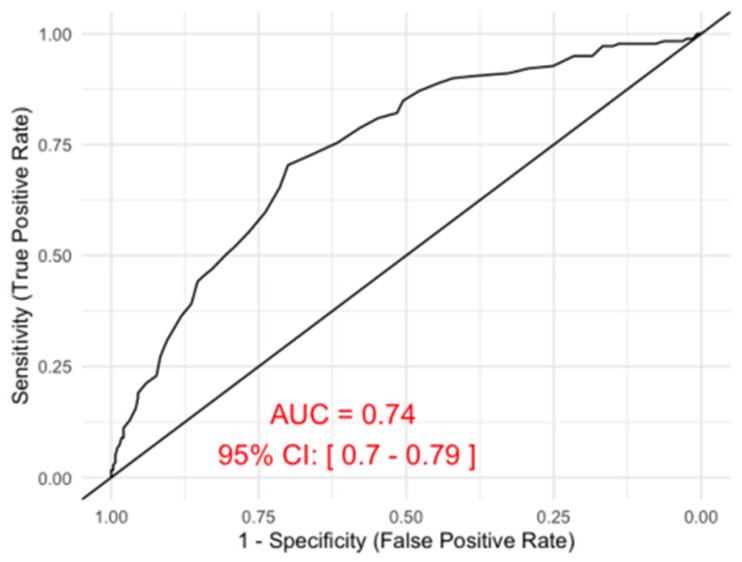
Receiver operation characteristic curve. AUC = area under the curve; CI = confidence interval.

The area under the curve (AUC) was 0.74, 95% confidence interval 0.70 to 0.79. (Figure 2) Using Youden’s J statistic as the criterion for selecting the optimal threshold, the best cut-off value was determined to be 13.35 g dL^−1^. At this threshold, the sensitivity was 70.4% and the specificity was 70.0%, while the negative predictive value (NPV) was 79.1% and the positive predictive value (PPV) was 59.4%.

### 3.4. Differences Between the Methods

In this study, we detected a notable bias between the two measurement methods, where SpHb measurements generally overestimated Hb by an average of 2.3 g dL^−1^ (95% CI 2.43 to 2.16).

**Figure 3:**
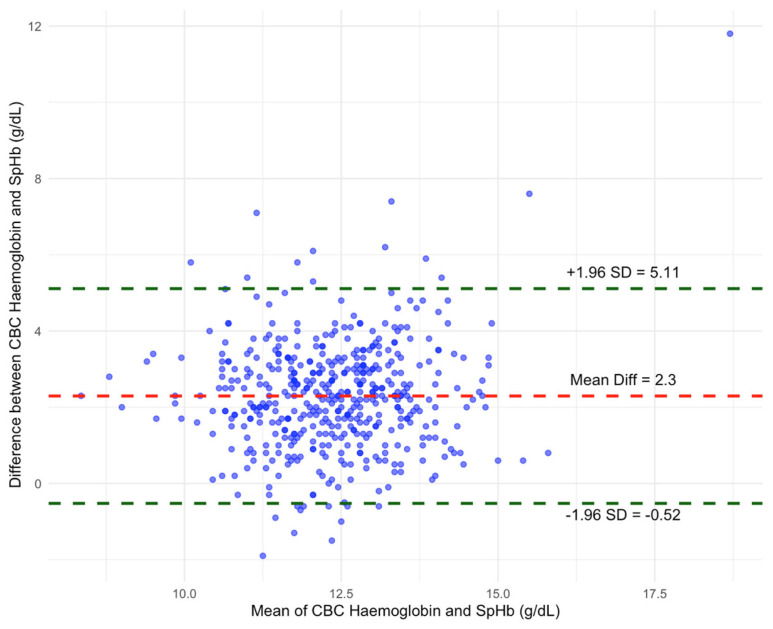
Bland–Altman plot. CBC = complete blood count; SpHb = non-invasively measured haemoglobin.

The standard deviation of the bias was 1.44, with a standard error of the mean (SEM) of 0.07. The limits of agreement (LOA) showed variation, with an upper LOA of 5.11 (95% CI: 4.89 to 5.34 and a lower LOA of −0.52 (95% CI: −0.29 to −0.75). This range indicated that, in many cases, SpHb can both overestimate and underestimate Hb; however, it demonstrated a greater tendency towards overestimation (Figure 3).

## 4. Discussion

### 4.1. Principal Findings

With a sensitivity of 5%—albeit good specificity (99%)—the SpHb measurement was unable to detect anaemia in most postpartum women in this study population. The AUC of 0.74 (95% CI 0.6955 to 0.787) and a wide range of LOA have further underlined the insufficiency of the device to produce consistently accurate measurements.

The wide LOA observed in the Bland–Altman analysis suggested that individual SpHb measurements may substantially over- or underestimate true haemoglobin values. From a clinical perspective, such variability limited the utility of SpHb for decision-making at the individual patient level, particularly in situations requiring precise assessment of anaemia severity.

With a threshold of 13.75 g dL^−1^, 145 (81%) anaemic women would have been successfully detected by SpHb measurement, but even with this adjustment, the device misclassified 34 anaemic women. The inclusion of a high number of women with postpartum anaemia and the attention to good perfusion (indicated by a PI > 2) during measurement supported the validity of our findings.

### 4.2. Interpretation in the Context of What Is Known

Numerous studies have been conducted to evaluate the efficacy of non-invasive Hb measurement devices to detect anaemia across different settings and various populations, ranging from blood donor clinics to intensive care units. These investigations have demonstrated that SpHb measurement devices can exhibit considerable variability in their accuracy for anaemia detection, with a relatively consistent trend towards lower sensitivity in females [13,14,15,19].

Nevertheless, only a limited number of studies have compared spot-check haemoglobin co-oximetry to CBC measurement in postpartum women and, to the best of our knowledge, no study has successfully established a clinically relevant cut-off for the detection of anaemia in this context [9,18].

The most comparable and recent study on this topic was published in 2023 by Mills et al., which compared SpHb measurement with laboratory analysis on the first day of postpartum, in part using the same device employed in this study. They reported that the Rad-67™ Spot-check Pulse CO-Oximeters^®^ overestimated Hb by 2.2 g dL^−1^ on average and had a low sensitivity when detecting anaemia in postpartum women (16.4%). Even after adjusting for fixed bias, the sensitivity was reported to be 78.0% with a specificity of 88.1%. These findings have correlated strongly with our own observations [18].

Mills et al. discussed the possible limitations of a single, poorly calibrated device. In our study design, we addressed this issue by utilising two different Rad-67™ devices in the operating room for caesarean deliveries and for the measurements at delivery room dismissal. There was no significant difference in the accuracy of the two devices. Thus, poor device calibration was not likely to be a relevant contribution to the device’s measurement accuracy.

Previous studies have found a correlation between the perfusion index and the accuracy of SpHb measurements [16,17]. We therefore excluded SpHb measurements with an PI < 2, a cut-off that has been proposed before. However, only three patients were excluded because of persistent PI < 2 after three measurement attempts [17].

### 4.3. Clinical Implications

The findings of this study can further support a cautious utilisation of SpHb values in this high-risk clinical setting, as a quality screening test should have high sensitivity. The very low sensitivity observed in this study (5%) indicated that the majority of anaemic women would not have been identified by SpHb measurement alone. If used as a standalone unadjusted screening tool, the risk of false-negative findings may lead to under-detection of clinically relevant postpartum anaemia and delayed therapeutic intervention.

Nevertheless, the consistent trend to overestimate Hb values by an average of 2.3 g dL^−1^ (95% CI 2.43 to 2.16) created the possibility to adjust the measurement for this bias and help clinicians in the evaluation of women at risk of having undetected anaemia, especially in situations where venous blood sampling may not be feasible. Systematic overestimation may partly reflect altered peripheral perfusion and rapid intravascular volume changes in the immediate postpartum period; however, even with a cut-off of 13.75 g dL^−1^ the number of missed anaemic women was too high for a reasonably accurate screening test.

Most guidelines have suggested measuring Hb within 24 to 48 h after childbirth in women who have experienced blood loss exceeding 500 mL, who were identified to have untreated anaemia during pregnancy, or who displayed symptoms of anaemia in the postpartum period [5,22,23]. With insufficient accuracy to detect anaemia in postpartum women, even with an adjustment for fixed bias, our data cannot support a change in this clinical practice (i.e., replacement of CBC with SpHb measurement).

However, in settings where CBC measurement may not be feasible due to financial constraints or a lack of equipment (i.e., in developing countries, out of hospital births), the cut-off proposed in this study could potentially still assist midwives and other healthcare workers in detecting postpartum anaemia. Yet the potential public health benefit of this approach remains uncertain in the absence of cost-effectiveness and/or implementation data.

Postpartum haemorrhage has remained a leading cause of maternal mortality, particularly in low-income countries, and identifying anaemia in this context is a crucial component of global efforts to reduce these mortality rates because it has been shown to triple mortality in this setting [24]. More specifically, postpartum anaemia affects 50–80% of women in developing countries, while in developed countries, the prevalence is less than 30% [25,26]. Therefore, compared to no Hb measurements at all, SpHb measurements could still be beneficial in these settings. While SpHb can offer practical advantages such as rapid availability and non-invasiveness, these must be weighed against the risk of diagnostic inaccuracy compared with laboratory testing.

### 4.4. Research Implications

To the best of our knowledge, this was the first study to define a clinically relevant cut-off for the detection of postpartum anaemia with SpHb measurements (Rad-67™ Spot-check Pulse CO-Oximeters^®^). However, further investigations are needed to evaluate the usability of the defined threshold in this specific context.

The proposed statistical calculation of a cut-off with a maximised specificity while meeting a minimum sensitivity of 80% used in this study has been chosen based on previous research and clinical usability [19].

While statistical approaches such as regression-based adjustment could theoretically reduce systematic measurement bias, the present analysis focused on defining a clinically pragmatic screening threshold. Future research should explore whether modelling approaches can enhance the discriminatory performance of non-invasive haemoglobin monitoring in postpartum populations, and whether population-specific calibration strategies may further improve clinical applicability.

Nevertheless, a cut-off with higher sensitivity to meet the intrinsic goal of a reliable screening test could be a subject for further investigations. Conversely, a higher SpHb cut-off could potentially lose its relevance in a clinical setting.

### 4.5. Strengths and Limitations

This study included 466 measurement pairs and therefore represented one of the largest cohorts in this setting. Additionally, to the best of our knowledge, this was the first study in the postpartum phase to propose a clinically relevant cut-off for the detection of anaemia. A high rate of anaemic women (n = 179, 38.4%) and the exclusion of measurements with low PI were further strengths of this project.

Exclusion was primarily related to the availability of SpHb measurements rather than clinical factors. Furthermore, the predominance of caesarean deliveries in the study cohort and the single-centre design may limit the generalisability of the findings, particularly to women following vaginal birth. Haemoglobin measurements were obtained in the immediate postpartum period, which may not fully reflect subsequent haemodynamic redistribution and delayed haemoglobin decline. As haemoglobin concentrations can further decrease within the first 24–48 h after delivery, diagnostic performance estimates of SpHb in this study may differ from those observed at later postpartum time points.

Additionally, it was not possible to report reliable measurements of suspected blood loss to correlate these numbers with the observed Hb measurements. Furthermore, the time of SpHb and CBC measurement in the operation room, directly after delivery, could be too early to represent a relevant blood loss during the procedure.

## 5. Conclusions

Non-invasive SpHb measurements via spot-check co-oximetry did not adequately detect anaemia compared to CBC haemoglobin values in the setting of early postpartum women. The device consistently overestimated haemoglobin values. Even after the calculation of an optimal cut-off to adjust for this bias, the number of undetected anaemic women remained high. Based on the present findings, SpHb measurements should therefore not be considered a substitute for laboratory haemoglobin testing in the early postpartum setting.

However, when used with caution, the proposed cut-off may still offer potential value in settings where alternative methods of haemoglobin assessment may be unavailable. Any potential use in low-resource environments should be regarded as exploratory and would require further validation in multicentre studies and real-world implementation settings.

Future research should focus on identifying strategies to improve screening sensitivity and evaluate the potential clinical impact of integrating non-invasive haemoglobin monitoring into postpartum screening approaches.

## Figures and Tables

**Table 1:** Patient characteristic with standard deviation. BMI = body mass index; SpHb = non-invasively measured haemoglobin; CBC = complete blood count; PI = perfusion index.

	Count	Standard Deviation
Patients	323	
Measurements	466	
Measurements at delivery room discharge	266	
Caesarean delivery	395 (85%)	
Age [years], mean	32	5.2
BMI, mean	30.2	5.5
SpHb [g⋅dL^−1^], mean	13.42	1.43
CBC [g⋅dL^−1^], mean	11.21	1.22
Mean difference SpHb-CBC	2.3	1.44
PI, mean	14.1	5.2

**Table 2:** Diagnostic characteristics of SpHb measurement compared to with CBC for the diagnosis of anaemia defined as Hb  <  11 g/dL. The small number of SpHb measurements < 11 g dL^−1^ reflects the overestimation bias of the device.

Measurements n = 466		SpHb		
		Hb < 11 g dL^−1^ n = 11	Hb ≥ 11 g dL^−1^ n = 455	
CBC	Hb < 11 g dL^−1^ n = 179	True positive n = 9	False negative n = 170	Sensitivity 5%
	Hb ≥ 11 g dL^−1^ n = 287	False positive n = 2	True negative n = 285	Specificity 99.3%
Prevalence 38.4%		Positive predictive value 81.8%	Negative predictive value 62.6%	Accuracy 63.1%

**Table 3:** Diagnostic characteristics of SpHb measurement with a threshold of 13.75 g/dL compared to CBC for the diagnosis of anaemia defined as Hb  <  11.0 g/dL.

Measurements n = 466		SpHb		
		Hb < 13.75 g dL^−1^ n = 275	Hb ≥ 13.75 g dL^−1^ n = 191	
CBC	Hb < 11 g dL^−1^ n = 179	True positive n = 145	False negative n = 34	Sensitivity 81%
	Hb ≥ 11 g dL^−1^ n = 287	false positive n = 130	true negative n = 157	Specificity 54.7%
Prevalence 38.4%		Positive predictive value 52.7%	Negative predictive value 82.2%	Accuracy 64.8%

## Data Availability

Original data and statistical code will be available upon reasonable request.

## Glossary

Hb	Haemoglobin
PPH	Postpartum Haemorrhage
WHO	World Health Organization
SpHb	Non-Invasive Hb Measurement
CBC	Complete Blood Count
NPV	Negative Predictive Value
PBM	Patient Blood Management
PI	Perfusion Index
ROC	Receiver Operating Characteristic
AUC	Area Under the Curve
